# Serological analysis reveals differential antibody responses between TB patients and latently infected individuals from the TB endemic country of Mozambique

**DOI:** 10.3389/fmed.2023.1286785

**Published:** 2023-10-09

**Authors:** Andy C. Tran, Eugenia Boariu, María García-Bengoa, Mi-Young Kim, Emil Joseph Vergara, Tufária Mussá, Rajko Reljic

**Affiliations:** ^1^Institute for Infection and Immunity, St George’s University of London, London, United Kingdom; ^2^Institute of Biochemistry, University of Veterinary Medicine Hannover, Hannover, Germany; ^3^Research Center for Emerging Infections and Zoonosis (RIZ), University of Veterinary Medicine Hannover, Hannover, Germany; ^4^LIONEX Diagnostics and Therapeutics GmbH, Braunschweig, Germany; ^5^Department of Microbiology, Faculty of Medicine, Eduardo Mondlane University, Maputo, Mozambique

**Keywords:** tuberculosis, latent infection, antibodies, serology, antigens, diagnostics, LTBI (latent TB infection)

## Abstract

Serological antibody profiling of tuberculosis (TB) patients and household contacts with latent TB infection (LTBI) could identify risk indicators of disease progression, and potentially also serve as an easily accessible diagnostic tool to discriminate between these two stages of *Mycobacterium tuberculosis* (*Mtb*) infection. Yet, despite significant efforts over many decades, neither application has yet fully materialised, and this is at least in part due to inconsistent and varying antibody profiles from different TB endemic regions. In this study, we conducted a retrospective exploratory analysis of serum antibodies in a cohort of active TB patients (ATB) and their interferon-gamma release assay (IGRA) positive household contacts (LTBI), as well as healthy controls (HC) from Mozambique, a country with a high TB burden from the Sub-Saharan region. Using several *Mtb* antigens as well as crude preparations of culture filtrate proteins (CFP) from *Mtb* and Bacille Calmette Guérin (BCG), we report that the most discriminatory response for TB and LTBI was observed for serum IgA antibodies to the MPT64 antigen, followed by IgG antibodies to Ag85B and CFP, with ATB patients having significantly higher levels than LTBI or BCG-vaccinated healthy controls. Conversely, sera from LTBI individuals had higher levels of IgG antibodies to the HBHA antigen than ATB. While our sample size (*n* = 21 for ATB, 18 for LTBI and 17 for HC) was too small to fully evaluate the diagnostic potential of these differing serological profiles, our study however preliminarily indicated high level of sensitivity (95%) and specificity (97%) of an ELISA MPT64-IgA test for discriminating TB from LTBI and healthy controls, supporting the notion that it alone, or possibly in combination with other antigens such as Ag85B or CFP could lead to development of an easily accessible diagnostic tool for TB.

## Introduction

The early and accurate diagnosis of TB is critical in the control and transmission of the disease. On the one hand, the rapid diagnosis of TB cases represents an essential factor in reducing morbidity and mortality rates. Isolating contagious individuals and promptly initiating effective chemotherapy reduces the patient’s symptoms, and the possibility of transmission is minimised. On the other hand, diagnosis of LTBI cases and identification of those at high risk of reactivation and progression to TB disease could diminish the reservoir of infection. However, TB disease can be challenging to diagnose, especially in children and those who have weakened immune systems. Additional tests beyond medical examinations are required. Initial tests are performed to detect immunoreactivity (skin test or blood IGRA), but further tests are required to establish the stage of the infection (sputum culture, chest x-rays), and the sensitivity of the mycobacteria to a particular treatment regimen (drug sensitivity tests or GeneXpert). Diagnostics based on blood tests are advantageous because they are easily accessible, rapid, and cost-effective. However, the World Health Organization (WHO) recommends against using current commercial serological tests for TB screening or diagnosis in endemic TB settings due to high variability in sensitivity and specificity, based on a commissioned and comprehensive meta-analysis by Steingart et al. (1). The available serological tests do not apply to the diagnosis of latent TB infection, extrapulmonary TB or paediatric TB. New and more accurate tests for TB diagnosis and alternative serological tests are therefore required. The current commercial serological tests differ in several of their features, including antigen composition and method of detection (2). However, 19 such serological tests were evaluated for specificity and sensitivity by a WHO study group, and it was found that they showed great variability and inconsistency (1 to 60% sensitivity and 53 to 99% specificity), thus recommending against their use (3).

Nevertheless, there is a renewed effort to improve on serodiagnosis of TB by either combining different tests (4), or combining multiple antigens or epitopes of *Mtb* (4–12), with some of the reports claiming discriminatory potential between active TB and LTBI (6, 7, 9, 10). In this study, we performed an exploratory study on a historic set of serum samples from TB patients, LTBI and healthy (BCG vaccinated) controls in Mozambique, collected between 2016 and 2018 as part of a wider study [(13, 14) the EMI-TB project]. We analysed antibody and isotype responses to several vaccine antigens as well as secreted culture filtrate proteins (CFP) of *Mtb* and BCG. While the sample size was not large enough to assess the full diagnostic value of any specific response, we nevertheless wanted to assess which antigens/antibody combinations could best discriminate between ATB and LTBI/HC, and which combinations correlate best on the individual patient level.

## Materials and methods

### Ethical statement

Studies on serum samples from TB patients and their contacts cohort in Mozambique were approved by the Ministry of Health Committee of Bioethics and Health 9ref 298/CNBS/15 as part of the EU Horizon 2020-funded project EMI-TB (643558). The serum samples were collected in the Maputo Region (Mozambique), using standard operating procedures, from TB patients and their contacts. The samples in this study were collected after receiving informed written consent from each participant over the age of 18 or through a guardian or parent for participants under 18 years old.

### Study subjects and clinical data

Participants were recruited between March 2016 and February 2018 at the Centro de Saúde de Mavalana and the Centro de Saúde da Machava II, both based in the Maputo region of Mozambique as part of a wider study (EMI-TB). The cohort included a total of 56 individuals, all were HIV negative and BCG vaccinated in childhood. Twenty one were diagnosed with pulmonary TB (TB), 18 were contacts classified as latent TB infected individuals (LTBI) and 17 were uninfected healthy contacts (HC). Participants in the LTBI and HC groups were contacts of TB cases in this study, and 89% were household contacts. Study participants were diagnosed using the QuantiFERON®-TB Gold in-Tube test (QFR-GIT) interferon-gamma release assay (IGRA) with a cut-off value for a positive test being 0.35 IU/ml, along with chest X-rays, GeneXpert MTB/RIF, sputum culture, acid fast bacillus/smear test and medical examinations for specific TB symptoms. The diagnostic criteria for ATB, LTBI and HC are described in Table 1. The serum samples from newly diagnosed pulmonary TB patients were collected prior to initiation of anti-TB treatment or within the first 5 days of treatment. TB contacts included individuals exposed to a microbiologically confirmed TB index case. Active TB disease was ruled out in IGRA positive contacts if they showed no clinical manifestation of the disease, a normal chest X-ray and negative microbiological readout. From LTBI and HC groups were excluded individuals with previous TB diagnosis or previous positive IGRA. Individuals who received anti-TB treatment before or receiving immunosuppressive treatment, patients with autoimmune disorders or HIV co-infected irrespective of CD4 count, end-stage renal disease diabetes, alcoholism, and pregnant women were excluded from the study.

**Table 1:** Diagnostic criteria for ATB, LTBI, and HC groups.

	ATB	LTBI	HC
Clinical manifestations	+	−	−
GeneXpert MTB/RIF	+	−	−
IGRA	+	+	−
Sputum culture	+	−	−
Sputum smear (acid fast bacillus test)	+	−	−
Chest X-ray	+	−	−

The demographics and baseline characteristics of the participants are summarised in Table 2.

**Table 2:** Demographic characteristics of the participants.

	ATB	LTBI	HC	Total
N	21	18	17	56
Gender	Male: 15 Female: 6	Male: 6 Female: 12	Male: 9 Female: 8	Male: 30 Female: 26
Age (Years) Median (95% CI)	28 (21–40)	29 (20–54)	15 (17–38)	25 (21–32)
Weight (Kg) Median (95% CI)	56.8 (45–59.6)	60 (50.5–71)	60 (52–66)	58.3 (52.4–61.3)
BMI Median (95% CI)	19.48 (18.22–20.26)	20.8 (16.88–26.19)	20.76 (18–24.79)	19.69 (18.99–21.93)
CD4 cells/μl Median (95% CI)	469 (292–714)	987.5 (834–1,219)	861 (685–959)	790 (657–901)
CD8 cells/μl Median (95% CI)	352 (236–470)	618 (431–760)	677 (435–815)	521.5 (417–629)

### Recombinant antigens

All recombinant antigens used in this study including Ag85B, ECH (ESAT6-CFP10), Acr/HspX, MPT64, HBHA, PE18, PE31, and PPE26 were provided by Lionex GmbH (Braunschweig, Germany), with PE18, PE31, and PPE26 provided by MGB (15) and the remaining antigens as part of the wider EMI-TB vaccine study (2015–2020). All antigens were expressed using the *E. coli* expression system, aside from MPT64 which was expressed in *M. smegmatis*, and purified to a high degree (>95%) of purity and made endotoxin free.

### Mycobacterial growth and culture filtrate preparation

*Mtb* H37Rv (cultured in a containment level 3 laboratory) and BCG Pasteur was grown for 3 weeks in Sauton media (HiMedia), a protein-free culture medium, in a static incubator at 37°C until an OD_600_ of 0.6 was reached. Bacterial cells were pelleted by centrifugation at 3000 RCF at 4°C, the supernatants harvested, then filter sterilised using 0.22 μm PES filters (Millex). After sterilisation, the culture filtrates (CF) were concentrated using a 3 kDa cut-off centrifugal concentrating unit (Amicon) at 2000 RCF and 4°C. The CF samples were then dialysed against PBS pH 7.4, at 4°C for 24 h using a Slide-A-Lyzer 3.5 kDa dialysis cassette (Thermo Fisher Scientific). The protein concentration of the CF samples was determined by measuring the optical density (OD_280_) against a PBS blank sample using a Jenway (Genova) spectrophotometer, and then aliquoted and stored at −20°C until further use.

### Serum ELISA

Patient blood samples were collected in serum separator tubes and the serum fraction was sterile filtered using 0.22 μm PES filters (Millex) prior to storage at −80°C. Antigen binding ELISA was carried out by coating ELISA plates (NUNC) with purified recombinant TB antigens or *Mtb*/BCG culture filtrate protein in coating buffer (3.03 g Na_2_CO_3_ + 6.0 g NaHCO_3_ in 1 L dH_2_O adjusted to pH 9.6) at 5 μg/ml and 50 μl per well overnight at 4°C. Plates were then washed three times with PBS and blocked with blocking buffer (PBS 5% (w/v) skimmed milk powder) for 2 h at room temperature. After three additional washes with wash buffer (PBS 0.01% (w/v) Tween-20) and serum samples were added at a 1:10 or 1:20 starting dilution and serial dilutions of 1:2 or 1:3 performed in blocking buffer followed by a 2 h incubation at room temperature. After three washes, relevant secondary antibodies (anti-human IgA, IgG, and IgM peroxidase-conjugated, all from Sigma) were then added at 1:1000 dilution and incubated for 1 h at room temperature. SigmaFast OPD (Sigma) was added after washing the plate 5 times, and absorbances read at 450 nm using a plate reader (Infinite 200Pro).

### Western blotting

20 μg of CF protein was loaded onto 9–12% BIS-TRIS gels (Invitrogen, Thermofisher Scientific), along with a reference ladder (BioRad) and run at 130 V. After electrophoresis, the proteins were transferred onto nitrocellulose membranes (GE) by means of the semi-dry transfer method using NuPage™ Transfer buffer (Novex, Life Technologies) supplemented with 10% (v/v) methanol. The transfer was performed at 20 V, constant current, for 40 min. After transfer, the nitrocellulose membranes were blocked with 5% (w/v) skimmed milk powder in PBS overnight at 4°C. The membranes were then washed three times with PBS and incubated with serum samples at 1:200 dilution in PBS 5% (w/v) skimmed milk powder for 2 h at RT with gentle agitation. After washing three times with PBS, the membranes were incubated with an anti-human IgG γ-chain specific horseradish peroxidase (HRP) conjugated antibody (Sigma) at 1:1000 dilution in PBS 5% (w/v) skimmed milk powder for 2 h at RT with gentle agitation. The membranes were developed with ECL Prime substrate solution (Amersham) after three washes and captured with a gel reader (Syngene G).

### Data analysis and interpretation

Serum ELISAs were plotted as Log_2_ end-point-titres (EPT) which was defined as the dilution at which the sample absorbance was 2-fold higher than background. Statistical significance was determined using the Kruskal-Wallis test with Dunn’s multiple comparisons test due to non-Gaussian distribution of data as determined by the D’Agostino & Pearson test. *p* values of >0.05 were considered statistically significant. A correlation matrix was performed using a two-tailed nonparametric Spearman correlation with a 95% confidence interval. Statistical analysis was performed using GraphPad Prism V10.

## Results

### Common vaccine antigens discriminate poorly between ATB and LTBI

**Figure 1:**
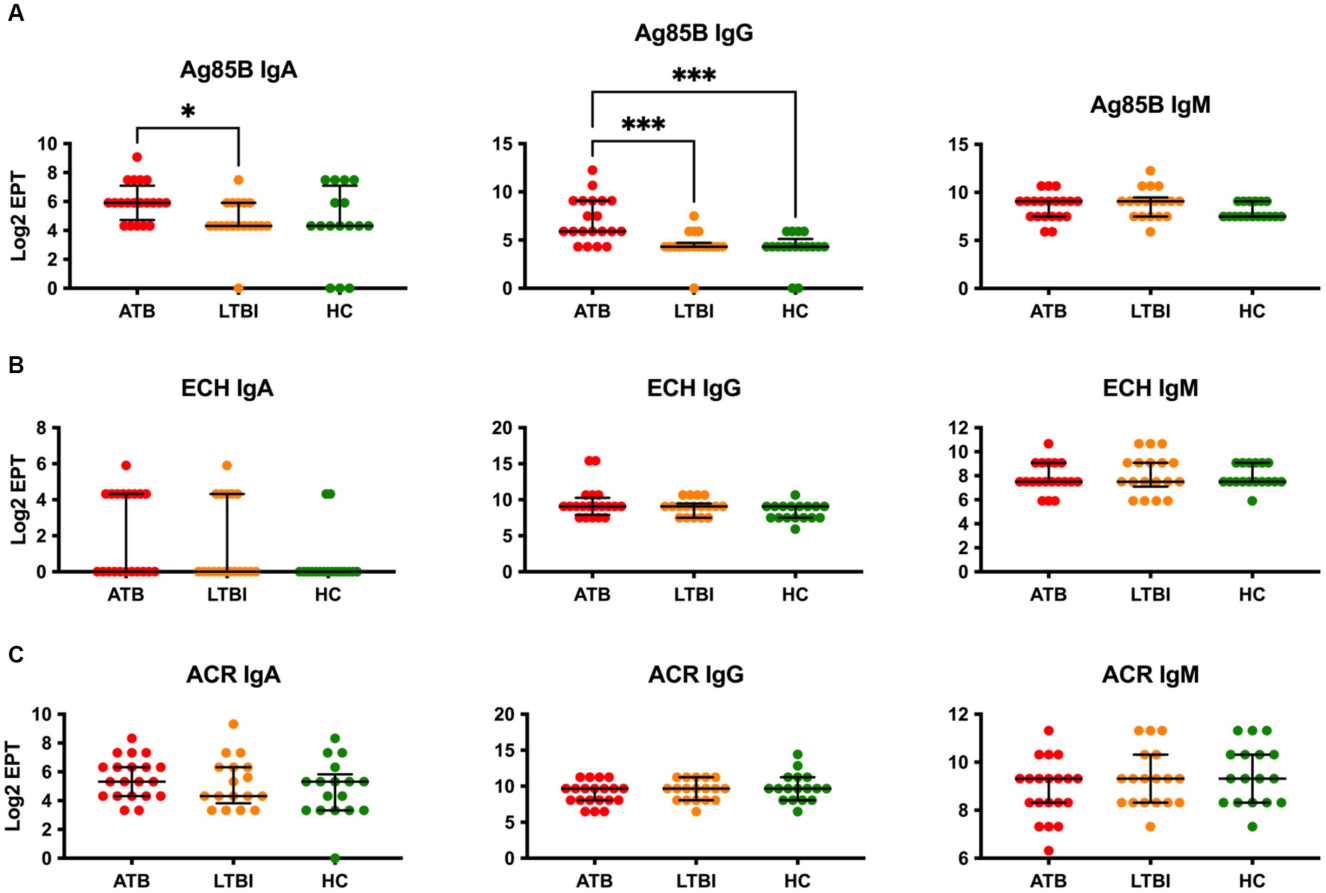
Serological IgA, IgG and IgM responses of study participants to common vaccine antigens Ag85B **(A)**, ECH fusion protein (ESAT6-CFP10) **(B)** and Acr/HspX **(C)**. EPTs were determined by ELISA and defined as a 2-fold absorbance value over background and presented as Log_2_ on a linear axis for ATB, LTBI and HC groups. Lines indicate median and IQR. Non-Gaussian distribution of the data was determined by the D-Agostino & Pearson test. Statistical significance was determined using the Kruskal-Wallis test with Dunn’s multiple comparison correction using GraphPad Prism V10. *p ≤ 0.05, ***p ≤ 0.001.

We first analysed serum antibody responses by ELISA in ATB, LTBI, and HC groups (who all were assumed to have received the BCG vaccination in childhood) in our Mozambique cohort using common TB vaccine antigens including Ag85B, ESAT6-CFP10 heterodimer (ECH) and Acr/HspX. Using endpoint antibody titres, we determined IgA, IgG and IgM responses to these antigens (Figure 1). The highest discriminatory capacity was demonstrated with IgG antibodies to Ag85B, with the difference between ATB and LTBI (Log_2_ EPT 5.907 vs. 4.322), or ATB and HC (Log_2_ EPT 5.907 vs. 4.322) being statistically significant (Figure 1A). However, neither IgA nor IgM antibodies to this antigen could discriminate between any of the groups (Figure 1A). Likewise, neither the ESAT6-CFP10 fusion protein (Figure 1B) nor the Acr antigen (Figure 1C) showed any discriminatory potential, with any of the three antibody isotypes tested.

### MPT64 IgA discriminates between ATB and LTBI

**Figure 2:**
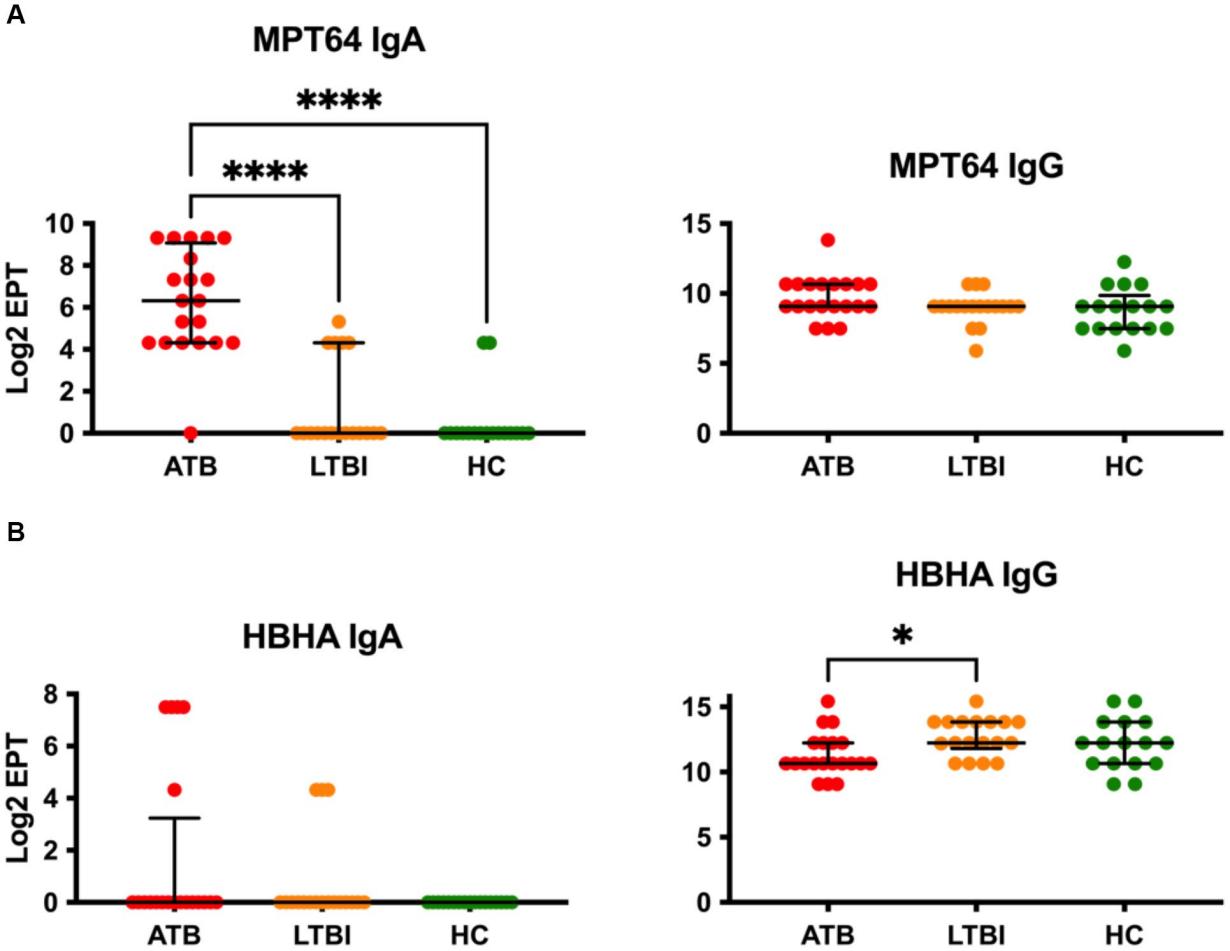
Serological IgA and IgG responses of study participants to Mtb antigens not present on BCG, MPT64 **(A)** and HBHA **(B)**. EPTs were determined by ELISA and defined as a 2-fold absorbance value over background and presented as Log_2_ on a linear axis for ATB, LTBI and HC groups. Lines indicate median and IQR. Non-Gaussian distribution of the data was determined by the D-Agostino & Pearson test. Statistical significance was determined using the Kruskal-Wallis test with Dunn’s multiple comparison correction using GraphPad Prism V10. *p ≤ 0.05, ****p ≤ 0.0001.

We next evaluated IgA and IgG responses to two further common vaccine antigens, namely MPT64 and HBHA. Remarkably, while IgG to MPT64 could not discriminate between any study groups, there was a highly statistically significant difference in IgA responses to this antigen (*p* ≤ 0.0001), with ATB displaying high titre responses while LTBI and HC being very low and indistinguishable from each other (Figure 2A). While intergroup variability of IgA responses in the ATB group for MPT64 was noteworthy (Log_2_ EPT IQR 4.322–9.072), nevertheless there was minimal overlap with the highest responders in the LTBI and HC groups. On the other hand, the IgA responses for HBHA antigen were low and indistinguishable between the three study groups, with only a small proportion of participants (4 of 20 for ATB and 3 of 18 for LTBI) showing a detectable response in our assay (Figure 2B). However, there was a statistically significant difference between the groups for IgG responses to HBHA, with LTBI participants displaying the highest titres (Figure 2B). Although statistically significant, the median difference between LTBI and ATB was relatively small (10.66 vs. 12.25), suggesting a poor discriminatory potential on its own. Interestingly, for both MPT64 and HBHA IgG responses, the HC group displayed similar responses to the ATB group, despite these participants not having been exposed to *Mtb* before.

### Serum antibody responses to PE/PPE family of *Mtb* antigens

**Figure 3:**
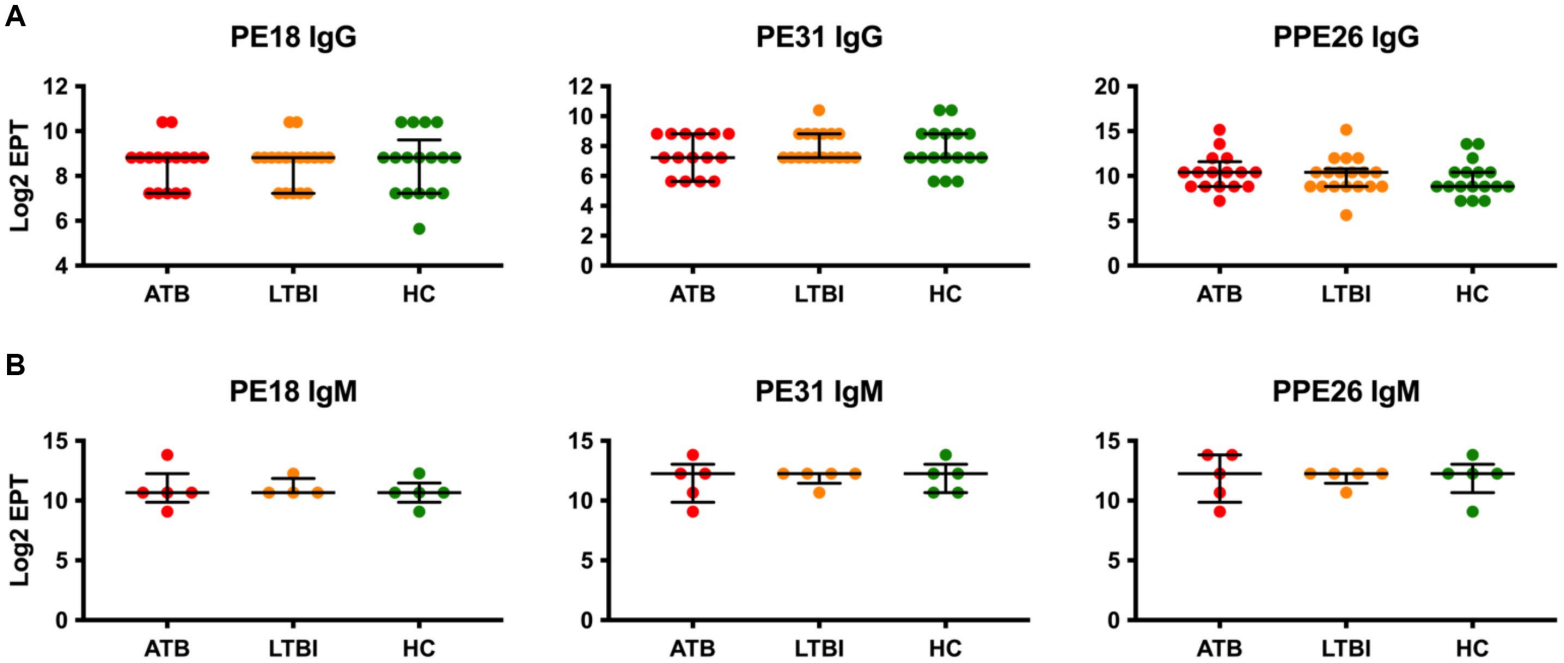
Serological IgG **(A)** and IgM **(B)** responses of study participants to PE/PPE family antigens, PE18, PE31 and PPE26. EPTs were determined by ELISA and defined as a 2-fold absorbance value over background and presented as Log_2_ on a linear axis for ATB, LTBI and HC groups. Lines indicate median and IQR. Non-Gaussian distribution of the data was determined by the D-Agostino & Pearson test. Statistical significance was determined using the Kruskal-Wallis test with Dunn’s multiple comparison correction using GraphPad Prism V10.

The PE/PPE family of proteins are highly represented in *Mtb*, contributing up to 10% of its genome (16). We therefore next investigated three members of this protein family, PE18, PE31, and PPE26, for IgG antibody responses in our cohort. Remarkably, while all three proteins were found to be immunoreactive, none showed any discrimination between the three groups of participants, with ATB, LTBI, and HC showing similar levels of IgG (Figure 3). While a small increase in median Log_2_ EPT of PPE26 IgG could be seen for ATB and LTBI individuals compared to HC, this difference was not statistically significant. Similarly, IgM responses in a subset (*n* = 5) of each group also showed no statistically significant differences between the three groups.

### Serum antibody responses to culture filtrate proteins of *Mtb*

**Figure 4:**
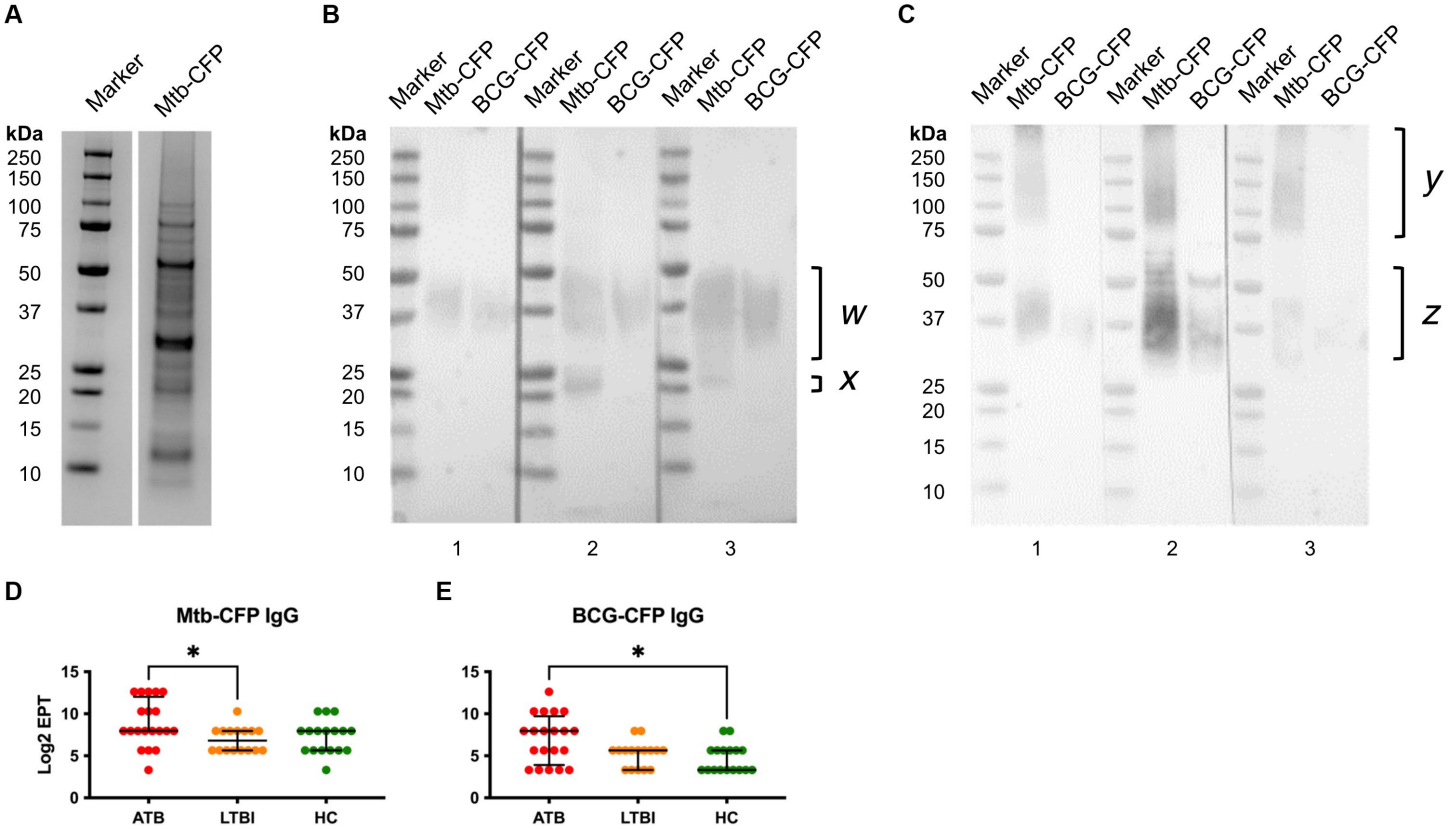
Characterisation of *Mtb*-CFP and sera responses to *Mtb* and BCG-CFP. *Mtb*-CFP was characterised by SDS-PAGE Coomassie stain **(A)** by comparing the protein to markers of known molecular weights. To determine reactivity of serum IgG from ATB patients **(B)** and LTBI individuals **(C)** to *Mtb*-CFP and BCG-CFP, a SDS-PAGE Western blot was performed on three representative patient samples in each group. Notable protein bands were present between 25-50kDa for *Mtb*-CFP (w) and BCG-CFP (z), bands corresponding to 20-25 kDa on *Mtb*-CFP, and a range of high molecular weight bands in LTBI samples (y). Serum IgG EPTs against *Mtb*-CFP **(D)** and BCG-CFP **(E)** were determined by ELISA and defined as a 2-fold absorbance value over background and presented as Log_2_ on a linear axis for ATB, LTBI and HC groups. Lines indicate median and IQR. Non-Gaussian distribution of the data was determined by the D-Agostino & Pearson test. Statistical significance was determined using the Kruskal-Wallis test with Dunn’s multiple comparison correction using GraphPad Prism V10. *p ≤ 0.05.

Since CFP proteins are likely highly accessible to B cells during TB infection, it is likely that they could induce strong antibody responses. Rather than testing individual known secreted proteins, we instead tested the whole CFP fraction, after liquid culture of *Mtb* or BCG. To avoid the presence of abundant bovine serum albumin in standard Middlebrook 7H9 media supplemented with OADC, *Mtb/*BCG was instead cultured in protein-free Sauton medium. The CFP were then filtered and concentrated for serological analyses of antibodies. We first analysed *Mtb*-CFP by SDS-PAGE and Western blotting, using representative serum samples from ATB and LTBI participants (Figure 4). SDS-PAGE revealed presence of a range of proteins in the *Mtb*-CFP fraction, ranging in size from 6 to >100 kDa (Figure 4A). Two prominent protein bands in particular could be observed at approximately 30 and 65 kDa. However, these proteins were not detected by Western blotting, by probing either with ATB or LTBI sera (Figures 4B,C). Instead, a prominent smeared band with a size of 30–60 kDa was recognised, along with a couple of distinct bands at approximately 20 and 25 kDa. In addition, sera from LTBI individuals also appeared to recognise high molecular weight proteins (>100 kDa), which were not recognised by sera from ATB individuals. These proteins were apparently not present in the BCG-CFP, as the same sera did not detect this protein band.

To compare the responses to *Mtb*-CFP by sera of all three study groups, we also performed an ELISA, which revealed significantly higher responses with ATB sera than LTBI group (Figure 4D). For BCG-CFP, sera from the ATB group showed significantly higher responses than the HC group (Figure 4E). Serum IgG responses against *Mtb* and BCG-CFP were not significantly different between LTBI and HC groups.

### Correlation analyses

**Figure 5:**
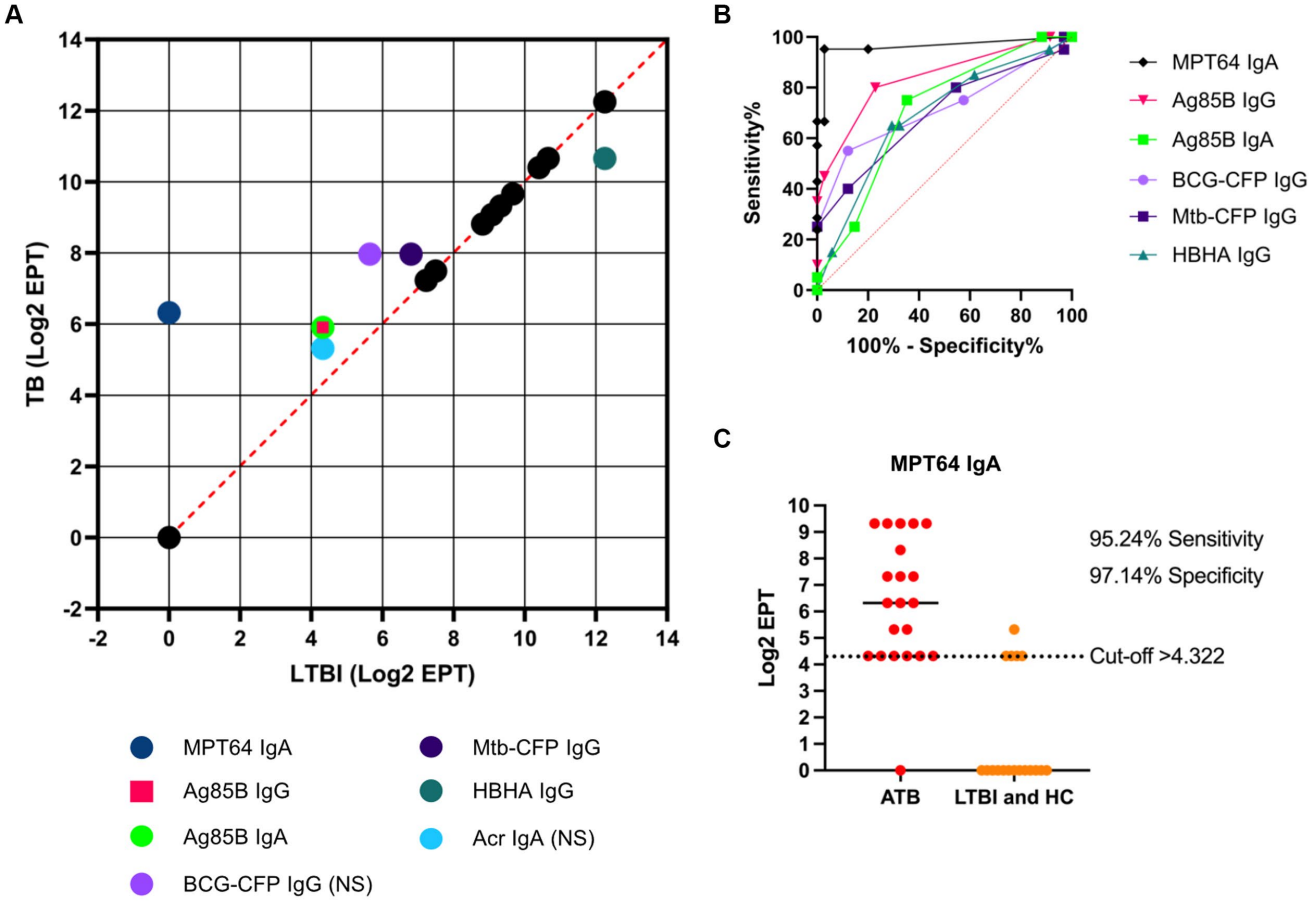
Diagnostic potential of serological responses to TB antigens. A correlation analysis was performed by plotting the median Log_2_ EPT for ATB and LTBI groups, with dashed orange line indicating R^2^ = 1 **(A)**. “NS” indicates that the difference between ATB and LTBI groups was not statistically significant (*p* > 0.05) as determined by using the Kruskal-Wallis test with Dunn’s multiple comparison correction. ROC analysis of serological responses with discriminating ability between TB and LTBI **(B)** with dotted orange line indicating AUC = 0.5. Illustration of the diagnostic potential of serum anti-MPT64 IgA **(C)**. EPTs were determined by ELISA and defined as a 2-fold absorbance value over background and presented as Log_2_ on a linear axis for ATB and LTBI + HC groups combined. Line indicates median. Data was analysed using GraphPad Prism V10.

The diagnostic potential of the serological responses to TB antigens and *Mtb*/BCG-CFP was visualised by plotting the median Log_2_ antibody EPT between ATB and LTBI groups (Figure 5A). Most antibody responses against the tested antigens between ATB and LTBI groups were highly correlated, indicating little to no discriminatory ability. However, we observed that IgA responses to MPT64 showed the largest discriminatory effect (an inverse correlation), followed by IgA responses to Ag85B and IgG responses to Ag85B, BCG-CFP, Acr/HspX and *Mtb*-CFP. While a difference in antibody titres was observed for IgG against HBHA, titres of anti-HBHA IgG were higher in LTBI participants than TB infected individuals. We further assessed the diagnostic performance of serological responses by determining the AUC (area under curve) of a ROC (receiver operating characteristic) curve for serological responses with the greatest discriminatory effect between ATB and LTBI groups (Figure 5B). Here, we found the greatest potential in IgA responses to MPT64 with an AUC of 0.9633 (95% CI 0.9018-1), followed by anti-Ag85B IgG (0.8393, 95% CI 0.7237–0.9548), anti-BCG-CFP IgG (0.7152, 95% CI 0.5590–0.8713), anti-*Mtb*-CFP IgG (0.6955, 95% CI 0.5421–0.8488) and anti-HBHA IgG (0.6890, 95% CI 0.5403–0.8376).

As an example of the diagnostic ability of IgA responses to MPT64, a Log_2_ EPT cut-off point of >4.322 was chosen, which resulted in a sensitivity of 95.24% (95% CI 77.33–99.76%) and specificity of 97.14% (95% CI 85.47–98.85%) for discriminating between ATB patients and LTBI/HC (Figure 5C).

**Figure 6:**
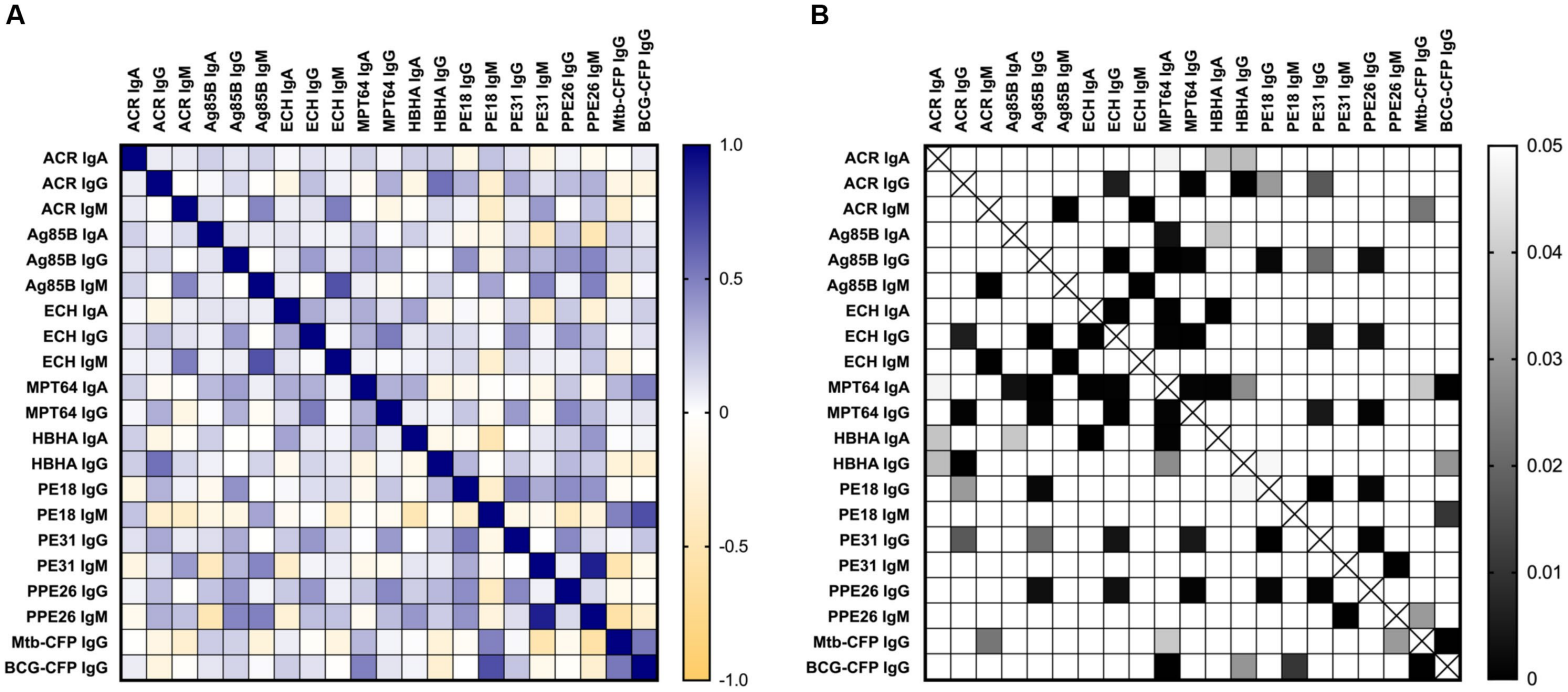
Correlation matrix for all serological responses tested against TB vaccine antigens and *Mtb*/BCG-CFP. A nonparametric two-tailed Spearman correlation matrix was performed **(A)** where blue represents a high R^2^ value (strong positive correlation), white indicates no correlation and orange represents a negative R^2^ value (strong negative correlation). The 95% confidence interval for each comparison was also plotted **(B)** with white boxes indicating *p* > 0.05 and progressively darker boxes indicating *p* values smaller than 0.05. Data was analysed using GraphPad Prism V10.

With a view of potentially combining several serological responses together to improve diagnostic performance, the correlation of all serological responses tested was performed on a patient level (Figure 6A). Here, a highly positive R^2^ value between two responses, indicating a strong positive correlation, suggests that individuals with high responses for one antigen/antibody isotype were likely to have high responses for the other, and vice versa. The patient-level data is important to inform the process of combining multiple serological tests, particularly if responses are negatively correlated. Anti-*Mtb*-CFP and anti-BCG-CFP IgG titres were highly correlated (R^2^ = 0.53), suggesting the presence of antibodies against antigens shared by *Mtb* and BCG. Notably, we found that IgA responses to MPT64 (the condition tested with the greatest diagnostic potential) correlates positively with IgG responses against Ag85B, BCG-CFP and *Mtb*-CFP (R^2^ = 0.37, 0.49, and 0.28 respectively), while negatively correlating with anti-HBHA IgG titres (R^2^ = −0.2), with these correlations being statistically significant (Figure 6B).

## Discussion

In this study, we investigated serological responses to a range of *Mtb* antigens in TB, LTBI and HC groups of participants from the Maputo region in Mozambique. We were particularly interested to identify antigens that could best discriminate between TB and LTBI, as a potential diagnostic tool. We found that the highest discriminatory potential was observed with IgA antibodies to MPT64 antigen, followed by IgG and IgA antibodies to Ag85B, and IgG against secreted culture filtrate proteins from BCG and *Mtb*.

Diagnostics based on blood tests such as lateral flow or capillary tube assays are advantageous because they are more suitable for implementation in clinical laboratory settings with limited facilities, are minimally invasive, rapid, and cost-effective. However, the WHO recommends against using current commercial serological tests for TB screening or diagnosis in endemic TB settings due to high variability in sensitivity and specificity (3). The available serological tests do not apply to the diagnosis of latent TB infection, extrapulmonary TB or paediatric TB. More accurate new tests for TB diagnosis and alternative serological tests are required; however, the sensitivity of the seven currently available serological tests was increased to 84% when the results of those tests were combined (17). The current commercial serologic tests differ in several of their features, including antigen composition (one or more antigens), antigen source (e.g., native or recombinant), chemical composition (e.g., protein, carbohydrate, or lipid), extend or manner of purification of antigen(s), and class of immunoglobulin detected (e.g., IgM, IgG, or IgA) (1). Thus, in a multiplex approach where a panel of 28 *Mtb* antigens were analysed using computational tools by multivariate statistics, classification algorithms, and cluster analyses resulted in a sensitivity and specificity of 90 and 80%, respectively, of the immunoassay (18).

However, most serological analyses of antibodies to *Mtb* focus on IgG, which are also the basis of the current serological tests (1). In our study, we extended the analysis of antibody responses to a limited set of antigens to also the IgA and IgM isotypes. We could not detect any significant differences in IgM responses for the six antigens we tested (Ag85B, Acr/HspX, ESAT6-CFP10, and the three PE/PPE proteins). However, IgA profiling revealed some distinctive readouts between ATB and LTBI and/or HC including Ag85B which confirms previous observations (19). Of particular interest in our study were responses to the MPT64 antigen. While the IgG responses to MPT64 were indistinguishable between ATB, LTBI and HC, the IgA response was remarkably elevated in ATB patients, with no significant differences between LTBI and HC. Using a relatively small set of samples in this study, serum MPT64 IgA responses could discriminate between TB and LTBI/HC with a 95.24% (95% CI 77.3–99.76%) sensitivity and 97.14% (95% CI 85.47–99.85%) specificity. Notably, this result exceeds the WHO/Foundation for Innovative New Diagnostics and New Dew Diagnostics Working Group of the Stop TB Partnership’s Target Product Profile diagnostic requirement of 65% sensitivity and 98% specificity for a diagnostic test (20).

MPT64 is an important secreted virulence factor of *Mtb* (21), that we have also previously identified as a protective antigen in our vaccine studies in mice (22). It is of interest to note that another study also reported strong discrimination between TB and LTBI based on MPT64 IgA antibodies in a similar size cohort from South Africa, giving further credential to the utility of this assay (7). However, in contrast to that study, we did not observe significant differences between TB and LTBI for anti-Acr IgA antibodies, despite the monoclonal IgA antibodies against that antigen showing therapeutic potential in our previous studies (23–26). Other antigens used in our study, including Ag85B, HBHA and secreted proteins (CFP) could also discriminate between ATB and LTBI albeit with lower accuracy. Therefore, IgA to MPT64 stands alone as the most promising single diagnostic tool from our study, although combining it with other antigens and/or antibody isotypes could further improve on it, which we have not investigated.

Interestingly, for both MPT64 and HBHA IgG responses, the HC group displayed similar responses to the ATB group, despite these participants not having been exposed to *Mtb* before, based on the diagnostic tests performed as part of this study (Table 1). While the MPT64 gene is absent in most strains of BCG (27), certain strains such as the Tokyo strain retain the MPT64 gene in addition to secretion MPT64 protein (28, 29), which may negatively impact the performance of diagnostic tests based on MPT64 in regions which utilise certain strains of BCG. The impact of BCG vaccination and exposure to environmental mycobacteria should also be considered for the choice of antigens to use in a serological test, especially in high-TB burden countries such as Mozambique or in geographical locations where the BCG vaccine is mandatory.

It should be noted that the recombinant HBHA protein used in this study was produced in the *E. coli* expression system and lacking the C-terminal post translational modifications present on native HBHA (30, 31). Previous studies have demonstrated that polyclonal antisera recognised both native and *E. coli* produced HBHA. However certain monoclonal antibodies can recognise epitopes of native HBHA, but not recombinant HBHA produced in *E. coli* (32). It is therefore possible that the use of HBHA produced in *M. smegmatis* which contains the relevant post translational modifications could result in different antibody reactivities and diagnostic performance than those presented in this study.

The PE/PPE family of proteins which play a key role in *Mtb* virulence represents approximately 10% of the *Mtb* genome (16) and PE18, PE31 and PPE26 have been recently evaluated for their potential as novel TB vaccine candidates in an unrelated yet unpublished study. Furthermore, PE18, PE31 and PPE26 share structural characteristics and features with other proteins in the PE/PPE family such as PE17 which have previously been shown to offer diagnostic potential (33, 34). However, in the present study, none of the studied PE/PPE proteins showed any discriminatory potential between the three groups of participants, with all showing relatively high levels of IgG antibodies. This is perhaps not surprising, considering that this family of proteins is highly represented in the genome of *Mtb* (16), and is also shared by many other pathogenic and non-pathogenic (environmental) mycobacteria. However, of particular interest in our study were also the crude CF proteins from *Mtb*, which showed second best discriminatory potential after MPT64 IgA. These exported proteins represent essential virulence factors, having roles in different pathways of bacterial survival mechanisms. Some of these play a role in enhancing the ability of *Mtb* to escape the defensive mechanisms developed by host cells. For example, Antigen 85 complex proteins are involved in the biosynthesis and assembly of the mycobacterial cell wall (9). Other secreted proteins affect directly or indirectly the local immune responses manipulating host cell pathways such as phagosome maturation, cell death, cytokine response, inhibition of peptide antigen presentation, etc. (10). One such secreted protein is the 6-kDa early secreted antigen target (ESAT6), which prevents or delays the onset of anti-mycobacterial adaptive immune responses (11).

Our analysis of CFP indicated a wide range of protein species ranging from 6 to 100 kDa in size, with two proteins of approximately 30 and 55–60 kDa being the most dominant by representation. However, these proteins appear poorly immunoreactive, and instead sera from ATB patients recognised a broad protein band at around 37–40 kDa, which could represent the immunodominant 38 kDa antigen of *Mtb*, and also a prominent band at approximately 20 kDa, which could represent the 19 kDa antigen. Interestingly, while the 38 kDa immunoreactive band was also detected by sera of LTBI, the 20 kDa protein was not, and instead these sera recognised a range of proteins of 75–250 kDa, not present in sera from TB patients. The composition of these proteins would be of further interest, as such distinct antibody profiles in LTBI may imply some role in controlling *Mtb* infection. This would require identification of these antigens that may serve as target for either TB vaccines or immunotherapy using monoclonal antibodies.

The limitation of our study is that our cohort was relatively small and therefore not representative of a wider TB population, including different geographic areas where factors such as differences in circulating strains of *Mtb* could play a role in TB disease (35). Furthermore, the high incidence of TB in Mozambique combined with the limitations of determining LTBI and HC based on IGRA test (36) makes it difficult to fully ascertain the TB negative status of participants. Therefore, these preliminary findings should now be validated on bigger cohorts that are representative of the wider TB population, including their IGRA+ and IGRA- household contacts. The further investigation of the predictive ability of serum antibody responses to various *Mtb* antigens for progression LTBI to ATB could also be investigated. Nevertheless, our study suggests that inclusion of antibody isotypes other than IgG in serological profiling of TB and LTBI may be the best way forward to harness the potential of antibodies in the serodiagnosis TB, and that serum IgA responses against MPT64 in particular, have the potential to be used as a rapid diagnostic tool for active TB.

## Data availability statement

The raw data supporting the conclusions of this article will be made available by the authors, without undue reservation.

## Ethics statement

The studies involving humans were approved by Ministry of Health Committee of Bioethics and Health 9ref 298/CNBS/15 as part of the EU Horizon 2020-funded project EMI-TB (643558). The studies were conducted in accordance with the local legislation and institutional requirements. Written informed consent for participation in this study was provided by the participants’ legal guardians/next of kin.

## Author contributions

AT: Methodology, Writing – original draft, Writing – review & editing, Formal Analysis, Visualization. EB: Methodology, Writing – review & editing, Formal Analysis. MG-B: Methodology, Writing – review & editing, Formal Analysis. M-YK: Methodology, Writing – review & editing. EV: Writing – review & editing, Methodology. TM: Writing – review & editing, Resources. RR: Conceptualization, Writing – original draft, Writing – review & editing, Formal Analysis, Visualization.
