# Supplementary material for: Serological analysis reveals differential antibody responses between TB patients and latently infected individuals from the TB endemic country of Mozambique
Source: Front Med (Lausanne). 2023 Oct 9;10:1286785. doi: 10.3389/fmed.2023.1286785 (PMC10591198; doi:10.3389/fmed.2023.1286785)
Supplement: Supplementary file 1 [file Table_1.DOCX]

**Supplementary Table 1. Median Log2 EPT and IQR of serological responses from patients in the Mozambique cohort.**

|  |  | **Log2 EPT** | | |
| --- | --- | --- | --- | --- |
|  |  | **25% Percentile** | **Median** | **75% Percentile** |
| **Acr IgA** | **TB** | 4.322 | **5.322** | 6.322 |
|  | **LTBI** | 3.822 | **4.322** | 6.322 |
|  | **HC** | 3.322 | **5.322** | 5.822 |
| Acr IgG | **TB** | 8.077 | **9.662** | 9.662 |
|  | **LTBI** | 8.077 | **9.662** | 10.85 |
|  | **HC** | 8.077 | **9.662** | 11.25 |
| Acr IgM | **TB** | 8.322 | **9.322** | 9.322 |
|  | **LTBI** | 8.322 | **9.322** | 10.07 |
|  | **HC** | 8.322 | **9.322** | 10.32 |
| Ag85B IgA | **TB** | 4.718 | **5.907** | 7.096 |
|  | **LTBI** | 4.322 | **4.322** | 5.907 |
|  | **HC** | 4.322 | **4.322** | 7.096 |
| Ag85B IgG | **TB** | 5.907 | **5.907** | 9.077 |
|  | **LTBI** | 4.322 | **4.322** | 5.907 |
|  | **HC** | 4.322 | **4.322** | 5.114 |
| Ag85B IgM | **TB** | 7.492 | **9.077** | 9.077 |
|  | **LTBI** | 7.492 | **9.077** | 9.077 |
|  | **HC** | 7.492 | **7.492** | 9.077 |
| ECH IgA | **TB** | 0 | **0** | 4.322 |
|  | **LTBI** | 0 | **0** | 4.322 |
|  | **HC** | 0 | **0** | 0 |
| ECH IgG | **TB** | 7.888 | **9.077** | 10.27 |
|  | **LTBI** | 7.888 | **9.077** | 9.077 |
|  | **HC** | 7.492 | **9.077** | 9.077 |
| ECH IgM | **TB** | 7.492 | **7.492** | 9.077 |
|  | **LTBI** | 7.492 | **7.492** | 9.077 |
|  | **HC** | 7.492 | **7.492** | 9.077 |
| MPT64 IgA | **TB** | 4.322 | **6.322** | 9.072 |
|  | **LTBI** | 0 | **0** | 4.322 |
|  | **HC** | 0 | **0** | 0 |
| MPT64 IgG | **TB** | 9.077 | **9.077** | 10.66 |
|  | **LTBI** | 9.077 | **9.077** | 9.077 |
|  | **HC** | 7.492 | **9.077** | 9.869 |
| HBHA IgA | **TB** | 0 | **0** | 3.241 |
|  | **LTBI** | 0 | **0** | 0 |
|  | **HC** | 0 | **0** | 0 |
| HBHA IgG | **TB** | 10.66 | **10.66** | 12.25 |
|  | **LTBI** | 12.21 | **12.25** | 13.83 |
|  | **HC** | 10.66 | **12.25** | 13.83 |
| PE18 IgG | **TB** | 7.229 | **8.814** | 8.814 |
|  | **LTBI** | 7.625 | **8.814** | 8.814 |
|  | **HC** | 7.229 | **8.814** | 9.606 |
| PE18 IgM | **TB** | 9.869 | **10.66** | 12.25 |
|  | **LTBI** | 10.66 | **10.66** | 11.85 |
|  | **HC** | 9.869 | **10.66** | 11.47 |
| PE31 IgG | **TB** | 5.644 | **7.229** | 8.814 |
|  | **LTBI** | 7.229 | **7.229** | 8.814 |
|  | **HC** | 7.229 | **7.229** | 8.814 |
| PE31 IgM | **TB** | 9.869 | **12.25** | 13.04 |
|  | **LTBI** | 11.45 | **12.25** | 12.25 |
|  | **HC** | 10.66 | **12.25** | 13.04 |
| PPE26 IgG | **TB** | 8.814 | **10.4** | 11.59 |
|  | **LTBI** | 8.814 | **10.4** | 10.79 |
|  | **HC** | 8.814 | **8.814** | 10.4 |
| PPE26 IgM | **TB** | 9.869 | **12.25** | 13.83 |
|  | **LTBI** | 11.45 | **12.25** | 12.25 |
|  | **HC** | 10.66 | **12.25** | 13.04 |
| Mtb-CFP IgG | **TB** | 7.966 | **7.966** | 12.03 |
|  | **LTBI** | 5.644 | **6.805** | 7.966 |
|  | **HC** | 5.644 | **7.966** | 7.966 |
| BCG-CFP IgG | **TB** | 3.902 | **7.966** | 9.707 |
|  | **LTBI** | 3.322 | **5.644** | 5.644 |
|  | **HC** | 3.322 | **3.322** | 5.644 |
